# Prevalence of active cytomegalovirus infection at diagnosis of ovarian cancer and during chemotherapy and subsequent changes in cognitive functioning

**DOI:** 10.1186/s12885-023-11566-y

**Published:** 2023-11-03

**Authors:** Rachel I. Vogel, Ashley E. Stenzel, Heewon Lee, DeVon Hunter-Schlichting, Erin Wesley, Locke D. Uppendahl, Melissa A. Geller, Heather H. Nelson

**Affiliations:** 1 Department of Obstetrics, Gynecology and Women’s Health, University of Minnesota, 420 Delaware Street SE, MMC 395, Minneapolis, MN 55455 USA; 2 Masonic Cancer Center, University of Minnesota, Minneapolis, MN USA; 3 Department of Family Medicine & Community Health, University of Minnesota, Minneapolis, MN USA; 4 Genetics, Cell Biology and Development, University of Minnesota, Minneapolis, MN USA; 5 Division of Epidemiology and Community Health, University of Minnesota, Minneapolis, MN USA

**Keywords:** Ovarian cancer, Cytomegalovirus, Viral Infection, Chemotherapy, cancer-related cognitive impairment

## Abstract

**Purpose:**

One of the most frequently reported effects of cancer and its treatments is cancer-related cognitive impairment (CRCI). Viral infections may affect inflammation and immune function and therefore may influence patient symptoms, including CRCI. The goal of this study was to describe the prevalence of cytomegalovirus (CMV) infections at diagnosis, during, and after chemotherapy in individuals with ovarian cancer and explore CMV infection at diagnosis with cancer-related cognitive impairment (CRCI) following chemotherapy.

**Methods:**

We recruited adults newly diagnosed with ovarian, primary peritoneal or fallopian tube cancer at a single academic cancer center into two prospective studies. In Study 1 (N = 71), participants provided blood samples at diagnosis. In Study 2 (N = 18), participants provided blood samples and completed symptom surveys before, during and after front-line adjuvant chemotherapy. Serum CMV DNA levels were assessed using digital PCR; >100 copies/mL of serum was considered positive for active CMV infection (CMV+). CRCI was measured using the Functional Assessment of Cancer Therapy – Cognitive Function (FACT-Cog) questionnaire. Changes in FACT-Cog scores were compared by CMV status at diagnosis using t-tests at each time point.

**Results:**

At diagnosis, 29.2% were CMV+ (28.2% in Study 1, 33.3% in Study 2). Following three cycles of chemotherapy (Study 2), CMV positivity rose to 60.0% and then back down to 31.3% after chemotherapy. We observed significant differences in CRCI following chemotherapy by CMV status at diagnosis.

**Conclusion:**

Our data suggest that active CMV infection is common among patients undergoing treatment for ovarian cancer and may contribute to symptoms of CRCI.

## Introduction

Ovarian cancer is the most lethal gynecologic malignancy. Surgery, in combination with chemotherapy, is the most common front-line treatment regimen, often resulting in prolonged side effects. Morbidity is high as patients typically experience multiple physical and emotional symptoms associated with the disease and its treatment. Therefore, there is a significant need to identify factors that may play a role in patient symptoms and prognosis, particularly those that might guide future research toward intervention strategies.

Numerous long-term effects of cancer and cancer treatment are known; one of the most frequently reported is cancer-related cognitive impairment (CRCI) [1]. CRCI research has largely concentrated on neurotoxicity associated with chemotherapy, often referred to as “chemo brain” or “chemo fog.” Nearly all individuals diagnosed with ovarian cancer are treated with platinum-based chemotherapies, which have been associated with cognitive impairment. Symptoms are usually mild to moderate and generally do not affect all areas of cognitive function. Importantly, not all cancer survivors experience CRCI [2], and among those who do, some experience only short-term decline while others report long-term concerns, even 5–10 years post-treatment [3]. These deficits can significantly affect quality of life [1] and persistent symptoms may prevent a patient from returning to their previous occupation and/or activities [4, 5].

Several prospective longitudinal studies evaluating CRCI prior to and up to one year after chemotherapy have reported decreases in cognitive functioning in approximately 17–50% of breast cancer patients, where most CRCI research has focused; this cognitive impairment is often experienced for months or years after treatment completion [6]. One longitudinal study reported approximately 20% of women with ovarian cancer exhibited cognitive impairment in at least one measured domain [7]. Because only a subset of patients experience CRCI, it is critical to determine risk factors so high-risk individuals can be identified for intervention.

Viral infections can affect cognitive function. For example, some individuals develop long haul symptoms following infection with the SARS-CoV-2 virus, including brain fog, highlighting the link between chronic inflammation and changes in cognitive functioning [8]. Prior to SARS-CoV-2, there was emerging evidence that acute viral infections contribute to cognitive decline [9], including cytomegalovirus (CMV). Cytomegalovirus (CMV) is a highly prevalent herpes virus infection in the United States, with 50% of the non-Hispanic white population seropositive by age 50 [10, 11]. Following primary infection, CMV may reactivate from latency in response to inflammation or immune suppression. Medically induced immune suppression, such as transplant, induces CMV reactivation. Solid tumor treatment with chemotherapy is also immune suppressive, however, only a few small studies have demonstrated that CMV positivity increases during chemotherapy for a number of patients [12–14]. History of CMV infection, as assessed by immunoglobulin G (IgG), has been shown to be associated with all-cause and all-cancer mortality in large cohort studies [15–17]. We demonstrated that CMV IgG in the presence of high inflammation at time of diagnosis with ovarian cancer was associated with poorer survival, suggesting that active infection may be occurring in individuals with ovarian cancer [18].

We sought to assess the prevalence of active CMV infection among individuals at the time of ovarian cancer diagnosis and then following chemotherapy by quantifying circulating cell free CMV DNA in serum (CMV DNAemia). Further, we wanted to explore whether active CMV infection at diagnosis was associated with CRCI following chemotherapy.

## Methods

To address this objective, we utilized samples from two prospective studies of individuals 18 years or older diagnosed with epithelial ovarian (ovarian, primary peritoneal, fallopian tube) cancer at the University of Minnesota. All participants provided written informed consent and both studies were approved by the University of Minnesota Institutional Review Board.

### Study 1: recruitment and procedures

Study 1 (IRB: 1610M96942) was a cross-sectional study which enrolled chemo-naïve individuals undergoing surgery for suspected ovarian cancer. Recruitment occurred between January 2017 and April 2021. Individuals who presented at the Gynecologic Oncology Clinic or were admitted to the inpatient gynecologic cancer service at the University of Minnesota Medical Center with either clinical, laboratory and/or imaging findings suspicious for ovarian cancer undergoing surgery were recruited by the study coordinator. Following informed consent, individuals provided blood samples prior to surgery. A total of 71 patients with pathology confirmed epithelial ovarian cancer were included in this analysis.

### Study 2: recruitment and procedures

Study 2 (IRB: 1605M87302) enrolled individuals following a new epithelial ovarian cancer diagnosis who were planning to undergo at least 3 cycles of chemotherapy. Recruitment occurred between January 2016 and December 2018. Exclusion criteria included: history of other primary malignancy other than non-melanoma skin cancer, previous exposure to chemotherapy, previous dementia, Alzheimer’s disease or other cognitive impairment diagnosis, history of stroke or serious head injury/brain trauma, and previous or current neurologic or psychiatric disorders (excluding depression and anxiety). Potentially eligible patients were approached at their post-operative visit; those interested in the study provided informed consent and medical record release forms and complete the baseline assessment. Participants were asked to complete symptom surveys and provide blood samples at four time points: prior to receipt of chemotherapy, after three cycles, following completion of front-line chemotherapy (window 0–90 days, median = 19 days), and six months later. Participants were compensated $20 at each time point for a total of $80. Data from 18 participants who completed at least two time points were analyzed.

### Measures

CMV DNA was quantified in serum samples using digital PCR (dPCR). DNA was extracted using the Qiagan QIAmp DNA Mini Kit. For Study 1, DNA was extracted from 200ul of serum, and a 254 bp region of the UL556 gene was targeted in a dPCR reaction as previously described [19]. For Study 2, DNA was extracted from 500ul of serum and a 72 bp fragment of the UL54 gene was targeted [20]. For both studies, a Biomark HD instrument for PCR amplification, signal capture and quantitation was used. Positive control DNA (Reference Material 2366a from the National Institute of Standards and Technology) and a negative template control were included in each run. Final dPCR data were reported as average copies/mL of serum.

In Study 2, participants reported subjective measures of cognitive functioning at each time point. Perceived cognitive function is more strongly associated with quality of life and is more sensitive to subtle changes in function than clinical measures [21]. Further, there are significant associations between neuroimaging metrics and subjective cognitive complaints in cancer patients [22–25]. Cognitive functioning symptoms were assessed using the Functional Assessment of Cancer Therapy – Cognitive Function (FACT-Cog) questionnaire. The FACT-Cog Version 3 is a validated and reliable 37-item measure designed to assess cognitive complaints over the past 7 days in cancer patients across four subscales: perceived cognitive impairments [range 0–72], impact of perceived cognitive impairments on quality of life [range: 0–16], comments from others [range: 0–16] and perceived cognitive abilities [range: 0–28] [26]. A higher score indicates better-perceived quality of life and cognitive functioning. The subscales are not combined together and the perceived cognitive impairments subscale score is recommended to be considered the primary score.

Demographic and clinical data regarding cancer diagnosis were abstracted from the medical record for both studies.

### Statistical considerations

Demographic and clinical characteristics for participants in each study were summarized using descriptive statistics. To describe the prevalence of CMV infection at each time point, CMV DNAemia status was summarized as negative (< 100 copies/mL), positive (100 + copies/mL), or high positive (1,000 + copies/mL). Changes in cognitive functioning were summarized as a continuous change from baseline (diagnosis) for each FACT-Cog subscale at each time point. The associations between CMV status at diagnosis (prior to chemotherapy exposure) and changes in cognitive function were conducted using t-tests at each time point. A sensitivity analysis was conducted to adjust for age using multivariable linear regression models; due to the small sample size and exploratory nature of the analysis, no other potential confounders were included. Data were analyzed using SAS 9.4 (Cary, NC) and p-values less than 0.05 were considered statistically significant.

## Results

Patients enrolled in both studies were on average 59 years old, primarily non-Hispanic white, and had advanced-stage disease (Table 1).

**Table 1:** Participant demographic and clinical characteristics by study

	Study 1 N = 71		Study 2 N = 18	
Characteristic	N	Median (Range)	N	Median (Range)
**Age, years**	71	59.0 (36–88)	18	58.5 (32–78)
	**N**	**%**	**N**	**%**
**Race**				
Asian	1	1.4	0	0.0
Black or African American	0	0.0	2	11.1
White	65	91.6	16	88.9
More than one race	1	1.4	0	0.0
Missing	*4*	5.6	0	0.0
**Ethnicity**				
Hispanic or Latino	1	1.4	14	77.8
Not Hispanic/Latino	65	91.6	4	22.2
Missing	5	7.0	0	0.0
**Cancer Stage**				
I	10	14.1	2	11.1
II	7	9.9	0	0.0
III	34	47.9	12	66.7
IV	20	28.2	4	22.2
**Histology**				
High-grade serous	47	66.2	12	66.7
Low-grade serous	4	5.6	1	5.6
Mucinous	3	4.2	0	0.0
Clear cell	10	14.1	1	5.6
Endometrioid	6	8.5	3	16.7
Mixed	1	1.4	1	5.6
Carcinosarcoma	0	0.0	1	5.6
**Neoadjuvant chemotherapy**				
No			13	72.2
Yes			5	27.8

**Fig. 1:**
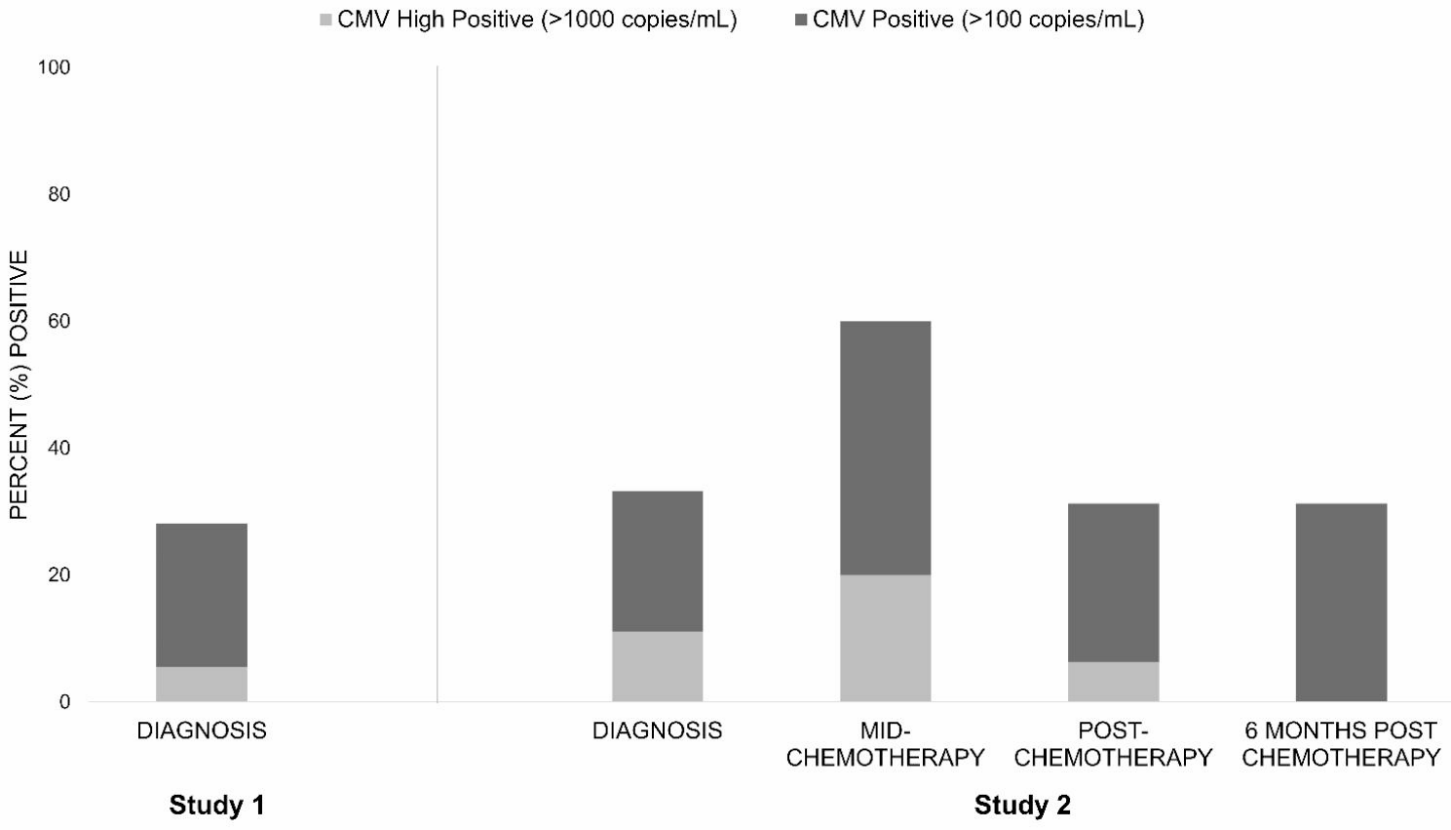
CMV DNA positivity among individuals with epithelial ovarian cancer at diagnosis and during front-line chemotherapy. Bar chart indicating the proportion CMV DNA positivity (> 100 copies/mL) and high positivity (> 1000 copies/mL) among participants with epithelial ovarian cancer at diagnosis (Study 1, n = 71; Study 2, n = 18) and during front-line chemotherapy (Study 2). The total height of the bar indicates the percent of participants who were CMV positive, with the light gray indicating the proportion with high positive CMV

Among the 71 participants in Study 1, 20 (28.2%) were CMV positive at the time of diagnosis; 4 (5.6%) were CMV high positive (Fig. 1). CMV DNAemia prevalence at diagnosis was similar in the 18 participants in Study 2: 6 (33.3%) were CMV positive and 2 (11.1%) were CMV high positive. Following three cycles of chemotherapy, CMV DNAemia prevalence rose to 60.0% (9/15) and then back down to 31.3% (5/16) and 31.3% (5/16) after chemotherapy and six months later, respectively. By the end of chemotherapy, 83.3% (15/18) of patients had tested CMV DNAemia positive at least once, with 55.6% (10/18) positive at more than one time point; 33.3% (6/18) were considered CMV high positive at some point since diagnosis.

**Fig. 2:**
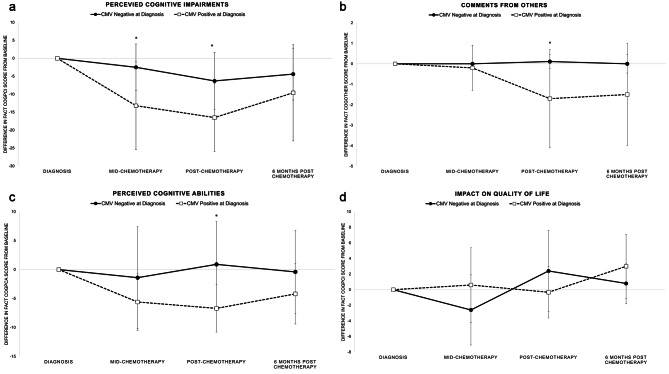
Differences in mean FACT-Cog subscale scores during and after chemotherapy for ovarian cancer by CMV status at diagnosis; Study 2, N = 18. FACT-Cog subscale scores calculated as differences from baseline (time of diagnosis). Positive CMV status at diagnosis is defined as CMV DNA positivity (> 100 copies/mL). Higher scores indicate better quality of life and cognitive functioning and therefore negative change indicates worse symptoms compared to baseline. Asterisk indicates p < 0.05

We observed significant changes in self-reported cognitive functioning from baseline (time of diagnosis) between those who were and were not CMV positive at diagnosis. Specifically, individuals who were CMV positive at diagnosis had greater decline in perceived cognitive impairments mid- (p = 0.027) and post-chemotherapy (p = 0.035) compared to those who were CMV negative (Fig. 2a). In addition, perceived cognitive abilities (p = 0.042; Fig. 2b) and comments from others (p = 0.046; Fig. 2c) were also more negatively affected post-chemotherapy among those who were CMV positive at baseline. No differences were observed on the impact of perceived cognitive impairments on quality of life subscale at any time point (Fig. 2d). Conclusions were similar after adjusting for age at diagnosis.

## Discussion

We observed that approximately one-third of individuals newly diagnosed with ovarian cancer were CMV positive at diagnosis and the majority were CMV positive at some point before or during chemotherapy. A small study of individuals with solid organ tumors undergoing chemotherapy similarly observed high rates of CMV reactivation and found viral loads to peak during the third cycle of chemotherapy [13]. While this phenomenon has been widely reported among patients receiving stem cell or solid-organ transplantations [27], the implications for patient symptoms among cancer patients receiving chemotherapy for solid tumors are largely unknown.

The association between CMV IgG levels and cognitive impairment has been reported in studies involving the general population [28–30] and cancer survivors [31] and support the hypothesis that CMV infection contributes to CRCI. While underlying mechanisms remain largely unknown, recent data suggest inflammation plays a key role in the development of CRCI related to chemotherapy. CRCI is multifactorial, and likely involves diverse and complex biochemical pathways. Several hypothesized mechanisms have been proposed, including an inflammatory response resulting in altered circulating cytokine profiles. Inflammation is involved in cognitive decline related to aging, neurodegenerative disorders, chronic illness, and surgery [32–35].

Much of the work done thus far exploring CMV among individuals with ovarian cancer has focused on CMV infection within the tumor [36–42], however few have looked at systemic infection. CMV is a major driver of both T-cell diversity and generalized inflammation. Primary CMV infection leads to enhanced inflammation which persists beyond the initial infection period [43, 44]. In addition, sub-clinical CMV reactivation from latency is associated with spikes in inflammation, which may contribute to patient symptoms, including CRCI after chemotherapy.

A strength of this analysis is the assessment of circulating cell free CMV DNA in serum as an indicator of active infection among individuals at the time of diagnosis and throughout chemotherapy rather than relying on IgG which does not reflect active infection. In general, these were low-level infections, and it is not clear what level of infection is clinically meaningful. Our threshold for CMV positivity represents the limit of quantification of the assay and reflects the threshold for CMV infection monitoring of bone marrow transplant recipients at our institution. Another limitation of this study is the small sample size, particularly for study 2. We therefore did not adjust for other potential confounding factors; these results are considered preliminary and hypothesis generating. Furthermore, data were obtained from a single academic institution in the Midwest and may not be generalizable.

## Conclusion

These data show that in some patients sub-clinical CMV infection is present at the time of ovarian cancer diagnosis and occurs during chemotherapy treatment for others. In addition, our data suggest that CMV infection at diagnosis may be associated with subsequent symptoms of CRCI, indicating a potential target for prevention. It is noteworthy that while participants with CMV infection at diagnosis reported more symptoms of CRCI, we did not observe differences on the subscale addressing whether the symptoms impacted their quality of life. We suspect the effect size was too small to be detected with this sample size, however, additional research is needed to examine this association in a larger sample. Further work in this area is warranted as there appears to be a substantial number of ovarian cancer patients with CMV infection at baseline and many that reactivate with treatment. It is unclear if CMV infection in particular is the culprit or if it is a biomarker for immune suppression. Regardless, the association of CMV with cancer mortality, its possible impact on patient symptoms, and available prevention and antiviral treatment strategies suggest the potential for high patient impact with further investigation in this population.

## Data Availability

The datasets generated and/or analysed during the current study are not publicly available due to the policies of the University of Minnesota Institutional Review Board and the details included in our patient consent forms, but are available from the corresponding author on reasonable request.

## Declarations

### Prior presentations

These data were presented in part by RIV as posters at the Society of Gynecologic Oncology Annual Meeting, Phoenix, AZ, March 18?21, 2022 and the Rivkin Center and the American Association for Cancer Research 14th Biennial Virtual Ovarian Cancer Research Seminar Series, September 2022.
